# Deep learning based prediction of extraction difficulty for mandibular third molars

**DOI:** 10.1038/s41598-021-81449-4

**Published:** 2021-01-21

**Authors:** Jeong-Hun Yoo, Han-Gyeol Yeom, WooSang Shin, Jong Pil Yun, Jong Hyun Lee, Seung Hyun Jeong, Hun Jun Lim, Jun Lee, Bong Chul Kim

**Affiliations:** 1 Department of Oral and Maxillofacial Surgery, Daejeon Dental Hospital, Wonkwang University College of Dentistry, Daejeon, Korea; 2 Department of Oral and Maxillofacial Radiology, Daejeon Dental Hospital, Wonkwang University College of Dentistry, Daejeon, Korea; 3 Safety System Research Group, Korea Institute of Industrial Technology (KITECH), Gyeongsan, Korea; 4 School of Electronics Engineering College of IT Engineering, Kyungpook National University, Daegu, Korea

**Keywords:** Machine learning, Translational research

## Abstract

This paper proposes a convolutional neural network (CNN)-based deep learning model for predicting the difficulty of extracting a mandibular third molar using a panoramic radiographic image. The applied dataset includes a total of 1053 mandibular third molars from 600 preoperative panoramic radiographic images. The extraction difficulty was evaluated based on the consensus of three human observers using the Pederson difficulty score (PDS). The classification model used a ResNet-34 pretrained on the ImageNet dataset. The correlation between the PDS values determined by the proposed model and those measured by the experts was calculated. The prediction accuracies for C1 (depth), C2 (ramal relationship), and C3 (angulation) were 78.91%, 82.03%, and 90.23%, respectively. The results confirm that the proposed CNN-based deep learning model could be used to predict the difficulty of extracting a mandibular third molar using a panoramic radiographic image.

## Introduction

A convolutional neural network (CNN) is a deep learning model that analyzes images and learns on its own^1^. In recent years, CNNs have been extensively used in many fields. In the healthcare industry, numerous studies have reported that a CNN can be used to analyze and diagnose medical images^1–7^. CNNs have also been used to better interpret the complexities of medical imaging by revealing patterns in large numbers of data and acquiring essential information to gain more knowledge^2^.

In the field of dentistry, CNNs have been applied for the detection of carious lesions, periodontal lesions, mandibular canals, cysts, and tumors^1,3,4,7–9^. They are also used to assess the difficulty of realizing endodontic treatment, skeletal classification, soft tissue profiling, osteoporosis, root morphology evaluation, and survival prediction of oral cancer patients^5,10–14^.

There are several potential complications that may arise after the extraction of a third molar, including pain, swelling, and nerve injury, and it is important to evaluate the difficulty of extraction in an objective manner^15,16^. In practice, clinicians often misjudge such difficulty and fail to complete the extraction process. The purpose of this study is to present a CNN-based deep learning model using panoramic radiographic images for predicting the difficulty of extracting mandibular third molars.

## Results

The classification results for the proposed diagnosis model are presented in Table 1. Here, C1 (depth), C2 (ramal relationship), and C3 (angulation) represent the three criteria of the Pederson difficulty score (PDS) used in this study^17^. The accuracies for C1, C2, and C3 were found to be 78.91%, 82.03%, and 90.23%, respectively. The accuracy and sensitivity for C1 were lower than that of the other criteria. Based on the C3 criterion, the data on score 4 were insufficient for the training and testing.

**Table 1:** Classification results.

Difficulty criterion	Score	Specificity (%)	Sensitivity (%)	Accuracy (%)	Kappa Score (%)
C1	1	92.05	88.13	78.91	70.88
	2	84.12	72.77		
	3	90.89	78.63		
C2	1	94.22	71.69	82.03	65.23
	2	69.52	90.73		
	3	98.29	61.36		
C3	1	92.67	94.15	90.23	85.54
	2	97.65	89.53		
	3	95.24	94.84		
	4	100*	0*		

*C1* depth, *C2* ramal relationship, *C3* angulation.

Superscript (*) indicates that the data in that particular case were insufficient.

**Figure 1:**
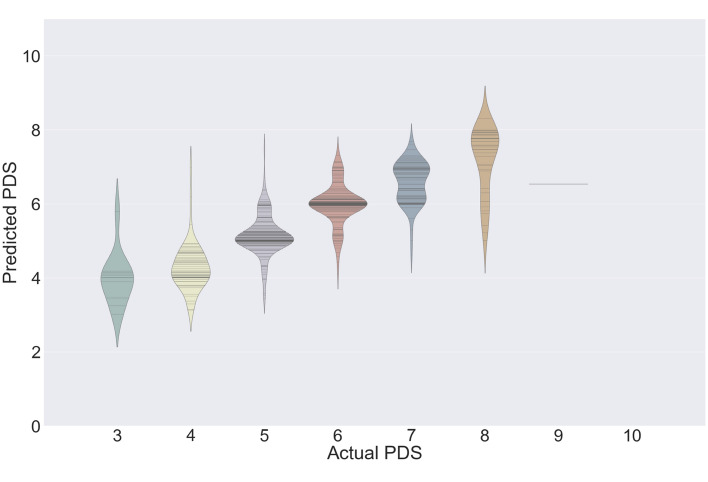
Predicted Pederson difficulty score (PDS) distribution of the actual PDS. The distribution of predicted PDS was generally close to the actual PDS. Whereas it performed well for PDS 4–7, it overestimated the cases of PDS 3 and underestimated the cases of PDS 8 and 9.

Figure 1 shows the distributions of the expected scores among the actual PDSs measured by medical experts. These scores are densely distributed near the actual PDSs. The distributions for scores of 9 and 10 were excluded because the data for these scores were extremely limited. The predicted PDS is calculated to consider the confidence distribution evaluated by the model, and the root mean square error (RMSE) between the expected PDS and actual PDS was estimated to be 0.6738. In addition, the Cohen’s kappa scores for each criterion were 70.88%, 65.23%, and 85.54%, respectively.

## Discussion

Deep learning has been widely used in various fields. Among the many deep learning models, CNNs are the most efficient^6^. CNNs have shown excellent results in the analysis of radiographic images when compared to the results by medical experts. Previous studies have shown that deep learning can be used to recognize anatomical structures, find anomalies, measure the distance, and classify structures in medical images^1,3–15^. However, in most studies, object detection was conducted manually, and tasks were limited to performing simple measurements, comparisons, or classifications. In this study, all processes were applied automatically including object detection. In addition, object detection is quite complicated because the normal anatomical structure is assessed and scored based on three criteria.

A Single Shot Multibox Detector (SSD) is a representative CNN-based model used for object detection. Liu et al*.* used this model to discretize the output space of the bounding boxes into a set of default boxes over various aspect ratios and scales^18^. This approach makes the training and integration of the detection system straightforward. Consequently, SSD shows a fast inference speed and achieves an outstanding detection performance.

We attached zero-padding to the edge to unify the image size during the preprocessing. The main reason for unifying the image size is to enable mini-batch learning. Notably, mini-batch learning not only speeds up the learning convergence it also increases the model efficiency. Meanwhile, ResNet, which is also a CNN, has delivered an excellent image recognition performance with residual learning implemented using skip connections.

**Figure 2:**
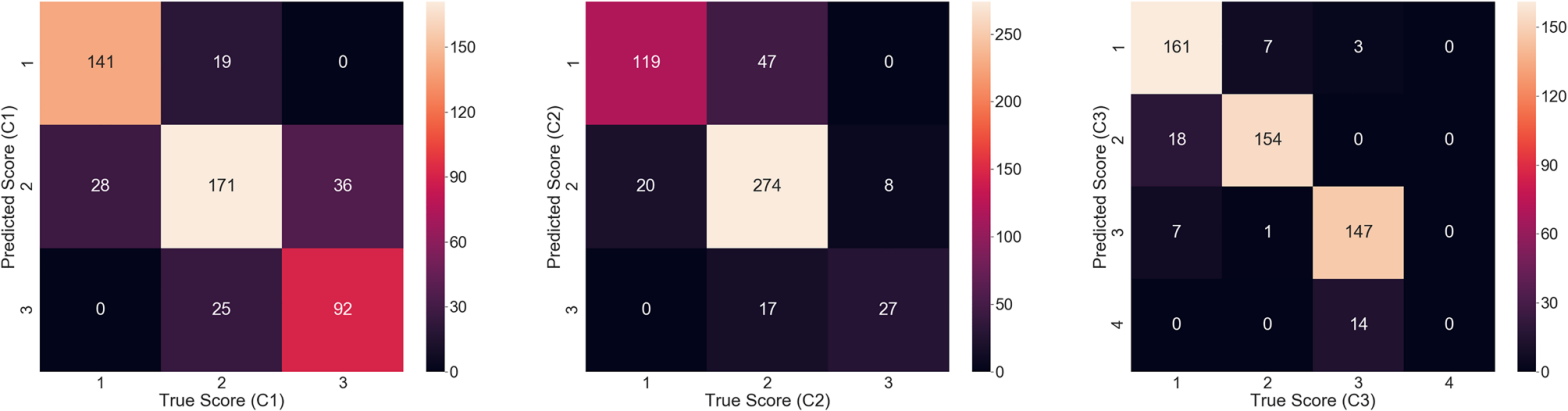
Confusion matrix showing the classification results for each criterion.

The experiment results are impressive. Figure 2 shows the results of the classification models according to each criterion as a confusion matrix. As the confusion matrix in Fig. 2 shows, there are a few cases in which the difference between the misclassified pairs of score is significant. Although a misclassification is a problem, if it does occur, the smaller the difference between the predicted and actual scores, the less significant the diagnostic error. Despite being unintentional, the above phenomenon is significant.

The primary objective of this study was to use a CNN to evaluate the difficulty of extracting mandibular third molars based on the features present in radiography images. Therefore, the correlation between the PDS evaluated by the proposed model and that measured by experts must be verified.

**Figure 3:**
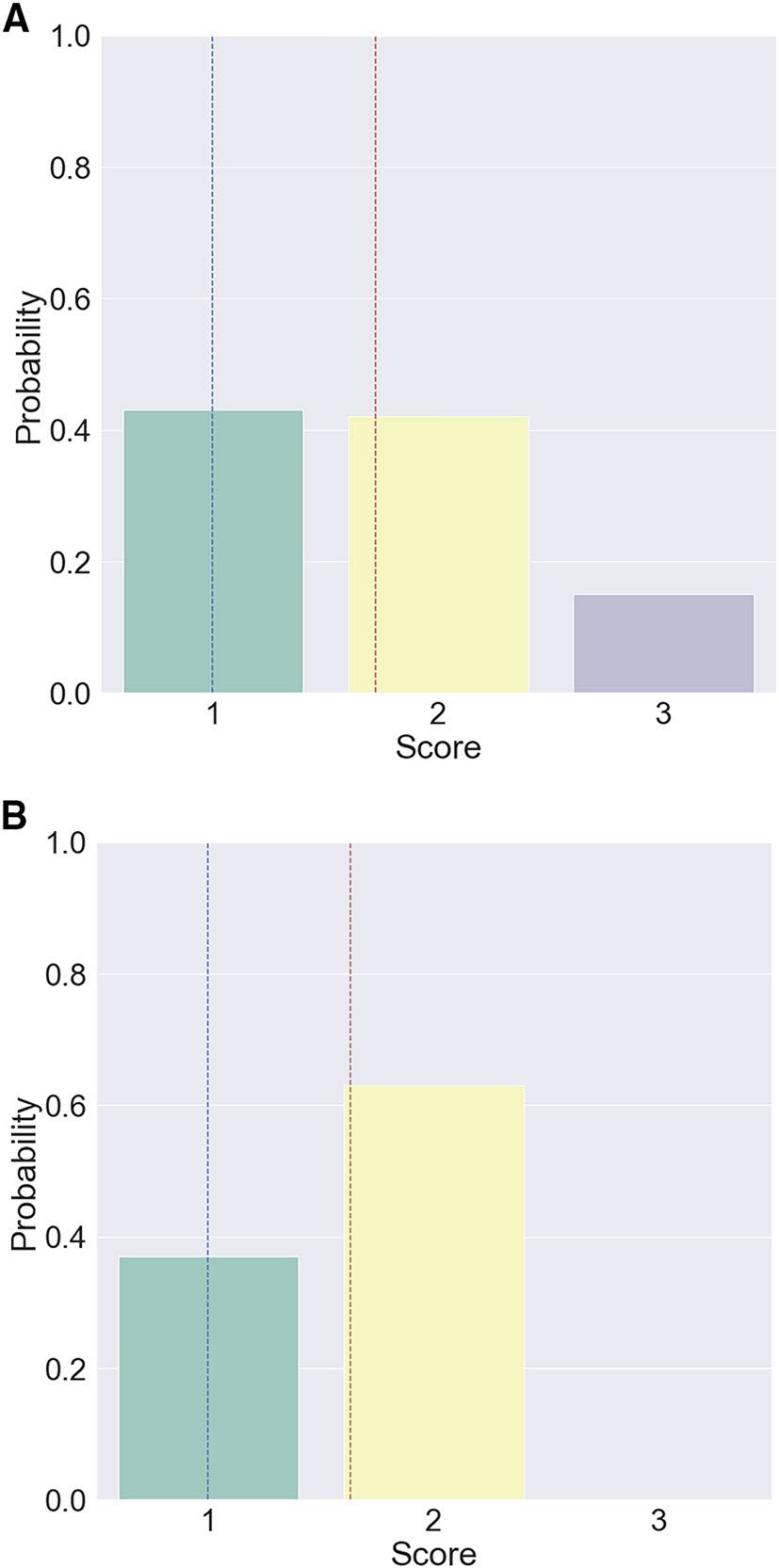
Example probability distribution inferred by the proposed model. The blue-dashed line indicates the actual score, and the red-dashed line indicates the expectation of the predicted PDS. If the model obtains a probability distribution as depicted in (**A**), it will be received a score of 1. Conversely, if a probability distribution similar to that depicted in (**B**) is obtained, its score will be miscalculated as 2.

In general deep learning based classifications, the class index with the largest value among the calculated class probabilities is selected. However, the data selected in this way may not accurately represent the data predicted by the model. For example, if the model obtains a probability distribution as depicted in Fig. 3 A, it will be received a score of 1. However, it can be seen that the model also has high confidence with of a score of 2. Conversely, if a probability distribution similar to that depicted in Fig. 3 B is obtained, its score will be miscalculated as 2; however, the probability for a score of 1 (the actual score) is also high. In the abovementioned cases, the classified scores cannot fully reflect the intention of the model. Therefore, we computed the predicted PDS based on the inferred probability distribution to reflect such intention. Given a probability $P_{{s_{c} }}$ for score $S_{c} \in \left\{ {1,2,3,4} \right\}$ of each criterion ${\text{c}} \in \left\{ {{\text{C}}1,{\text{ C}}2,{\text{ C}}3} \right\}$, the predicted PDS $\hat{y}$ can be calculated as follows:

$$\hat{y} = \mathop \sum \limits_{c} E\left[ {s_{c} } \right] = \mathop \sum \limits_{c} \mathop \sum \limits_{{s_{c} }} P_{{s_{c} }} \cdot s_{c}$$

In this way, the results can reflect the intention of the model.

The results show that accurate predictions of mandibular third molar extraction can be achieved using a CNN. However, although it performed well for scores of 4 through 7, it overestimated the cases of PDS 3 and underestimated the cases of PDS 8 and 9 (Fig. 1). Because PDS 3 is the lowest, a CNN can only estimate cases of PDS 3 or higher, leading to overestimated results on average. A similar occurrence was shown in cases of PDS 8 and 9. In addition, because there are no cases for PDS 10 and only two cases for PDS 9, one of which was used for testing and not learning, there has been little opportunity for the CNNs to learn about such cases and therefore CNNs have little information about them. This has led to an underestimation of the cases of PDS 8 and 9. It is likely that there are so few cases for PDS 9 and 10 because teeth with high scores for all criteria are rare. For example, a tooth with a vertical or distal angle (C3, with a score of 3 or 4) will not be interfered with by the adjacent mandibular second molar, and thus the score for C1 would be 1 or 2.

To the best of our knowledge, this is the first study on evaluating the difficulty of extracting a mandibular third molar using a deep learning model. These predictions will help the operator plan and prepare in advance, prior to the extraction process. The prediction results can also be used to inform patients about their conditions and seek their consent. In addition, objective data can be used to determine the treatment cost for extraction based on the level of difficulty.

There is a limitation however, in that we only used panoramic images. Panoramic images can show a broad range of anatomical structures in a single 2D image, although inevitable distortions occur in both the vertical and horizontal dimensions^19^. In addition, it is extremely difficult to evaluate a transverse angulation or dilaceration.

Clinically, there are many other factors that can affect the difficulty of extracting mandibular third molars; these include the gender, age, root morphology, bone density, and proximity to the inferior alveolar nerve^15,16^. Some studies have previously suggested that deep learning models can be used to evaluate certain factors related to the extraction difficulty. Hiraiwa et al*.* showed that CNNs can assess the rough morphology of the root of the mandibular first molar using a panoramic image^14^. This approach can be applied to the mandibular third molar, although more studies on evaluating the detailed morphology of the root, such as a dilaceration or partial curvature will be needed. Lee et al*.* showed that osteoporosis can be detected by analyzing the textural and morphological features in panoramic images using a CNN^13^. By analyzing the bone around the mandibular third molar and quantifying it, it will be possible to determine how much the bone density will affect the difficulty of extraction when using a panoramic image. For proximity to the inferior alveolar nerve, many previous studies have shown that CNNs can detect the inferior alveolar nerve using panoramic images and con beam computed tomography ^2,3,20^. It is possible to calculate the distance and location relationship between the inferior alveolar nerve and mandibular third molar using a CNN. However, there are no standardized variables for evaluating the difficulty of mandibular third molar extraction based on the distance or location. Further studies to quantify and standardize such variables and finally synthesize them will be needed.

## Materials and methods

### Datasets

A total of 600 panoramic radiographs of patients who underwent mandibular third molar extraction at the Department of Oral and Maxillofacial Surgery of the Wonkwang University Daejeon Dental Hospital in 2019 were randomly selected retrospectively (mean age of 27.5 years, standard deviation of 9.09, age range of 16–73 years, and 305 males and 295 females). Only patients with at least one fully developed mandibular third molar adjacent to an intact mandibular second molar were included. Patients who had severe periodontal disease or any other intraosseous disease that can affect the extraction were excluded.

The panoramic radiographs of the patients were obtained using a PCH-2500 (Vatech, Hwaseong, Korea) or ProMax (Planmeca, Helsinki, Finland) according to the user manual (72 kV, 10 mA, 13.5 for Vatech, 72 kV, 12 mA, 15.8 for Planmeca).

The 600 panoramic images included images of 1053 mandibular third molars. Each tooth was scored based on three criteria—depth, ramal relationship, angulation—according to the Pederson scale (Table 2). Scoring was applied with the consensus of three dentists, i.e., one oral and maxillofacial surgeon, one oral and maxillofacial resident, and one oral and maxillofacial radiologist, using two CX50N monitors (WIDE Co., Hwaseung, Korea). Because there was no precise boundary between the scores, the observers were calibrated as described below.

1. *Depth (C1)* The midpoint of an occlusal surface of the impacted third molar was set as the evaluation point. When the evaluation point was above the occlusal surface of the mandibular second molar, we recorded the score as a 1, and when it was below, we recorded it as a 2. When the entire tooth was below the occlusal surface of the mandibular second molar, we recorded the score as a 3.
2. *Ramal relationship (C2)* In mesio-angulation and horizontal angulation cases, a contact point of the mandibular third molar and the mandibular ramus was set as the evaluation point. The evaluation point was compared with the distal point of the cemento-enamel junction of the mandibular third molar. When the contact point was disto-apical, we recorded the score as a 1. When the contact point was mesio-occlusal, we recorded it as a 2. In the vertical and distoangular cases, we used the same points but only considered the occluso-apical position. When the contact point was apical, we recorded it as a 1. When the contact point was occlusal, we recorded it as a 2. Those scores for cases in which the entire crown was impacted were recorded as a 3.
3. *Angulation (C3)* The occlusal surface of the mandibular third molar was compared with the distal surface of the mandibular second molar. When they were close to perpendicular, we recorded the score as a 3, and when they were close to parallel, we recorded the score as a 2; otherwise, we recorded the score as a 1. Finally, we scored those cases with an angle of below 90° as a 4.

**Table 2:** Pederson scale used in this study for an evaluation of the difficulty of extraction.

Difficulty criterion	Classification standard	Score
Depth (C1)	Level A: high occlusal level	1
	Level B: medium occlusal level	2
	Level C: deep occlusal level	3
Ramal relationship (C2)	Class 1: sufficient space	1
	Class 2: reduced space	2
	Class 3: no space	3
Angulation (C3)	Mesio-angular	1
	Horizontal/transverse	2
	Vertical	3
	Distoangular	4

Level A: the occlusal surface of the mandibular third molar is at the same level as that of the occlusal surface of mandibular second molar. Level B: the occlusal surface of the mandibular third molar is between the occlusal surface and the cemento-enamel junction of the mandibular second molar. Level C: the occlusal surface of the mandibular third molar is below the cement-enamel junction of the mandibular second molar.

Class 1: there is sufficient space between the mandibular ramus and mandibular second molar for the crown part of the mandibular third molar. Class 2: space between the mandibular ramus and mandibular second molar is insufficient for the crown part of the mandibular third molar. Class 3: almost the entire crown of the mandibular third molar is impacted in the mandible.

To draw an objective conclusion, every score was cross verified. In the case of a disagreement, we followed the majority opinion. Subsequently, the PDS was determined as the sum of all scores obtained from each criterion^17^. Each radiograph was manually labeled by drawing rectangular bounding boxes around the mandibular third molars for region of interest (ROI) detection training.

### Preprocessing and composition

**Figure 4:**
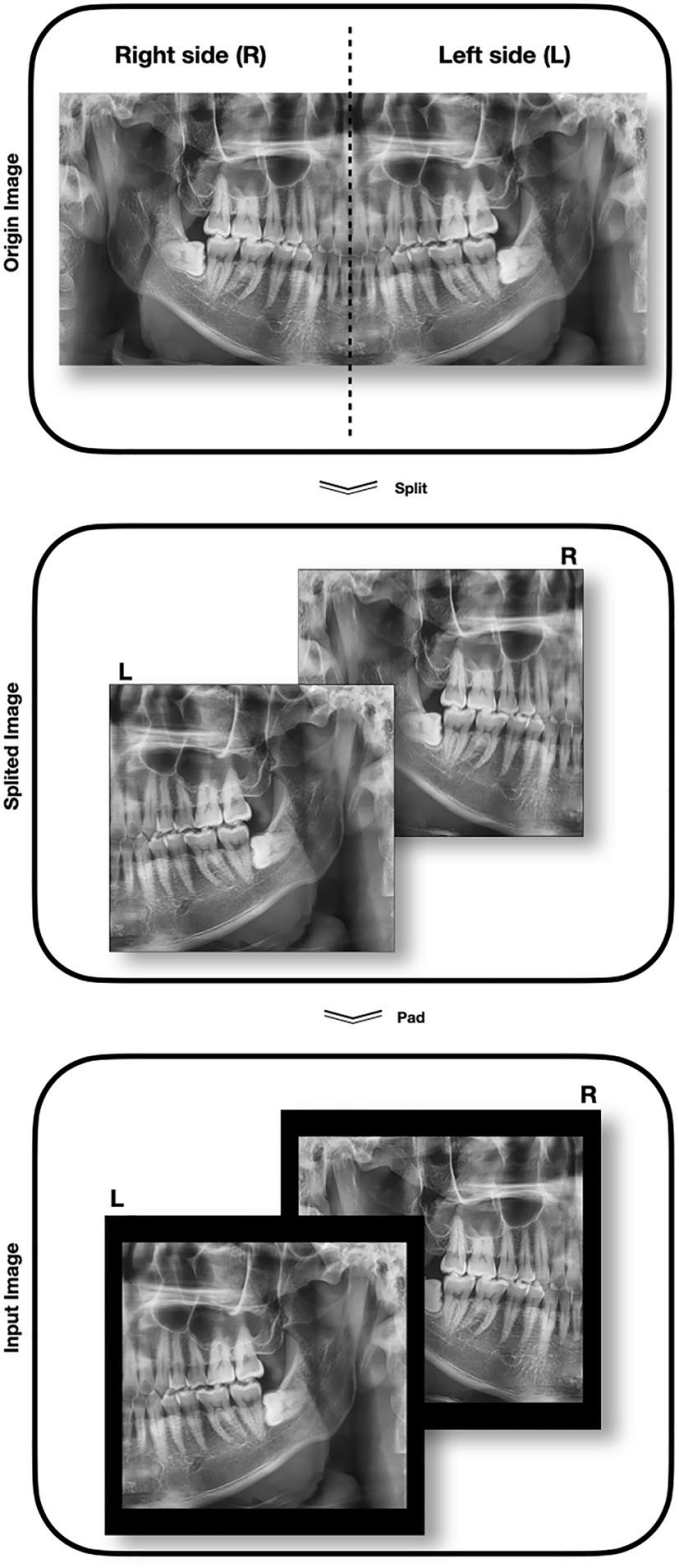
Preprocessing of panoramic images.

Preprocessing was required before the acquired images could be used for learning and verification. Figure 4 shows the preprocessing process. First, the original image was split into two sections (left and right) at the same ratio based on the width of the panorama image. The second image in Fig. 4 is the split image. Next, the edges were zero-padded to unify them at the same size. The sizes of the panoramic images obtained were different because the field of view varied slightly depending on the sizes of the objects. After pre-processing, the whole data were randomly sampled at a ratio of 1:1 according to the subject, and the sampled data were used as a training and testing set, respectively. The dividing process was performed only once at the first. And then, all of the experiments we had done were used the same dataset. As for the data for validation, 10% of data was reassigned from the training set. Learning the model and finding the optimal hyperparameters were done on the trainset and validation set. Only the finally selected model was evaluated on the test set and presented in this paper.

### Augmentation

Augmentation prevents an overfitting and helps in the learning of various features. As the augmentation techniques, we employed random flipping and rescaling in our detection model. The image was flipped with a probability of 0.5, and the scale was randomly converted within a ratio range of (0.8, 1.0). The brightness and contrast variation factors were randomly selected within the range of (0.8, 1.2). In addition, the ROI was randomly cropped from the entire image within a ratio range of (0.9, 1.0). All transformations for augmentation were applied differently for each iteration.

### Proposed diagnosis model

**Figure 5:**
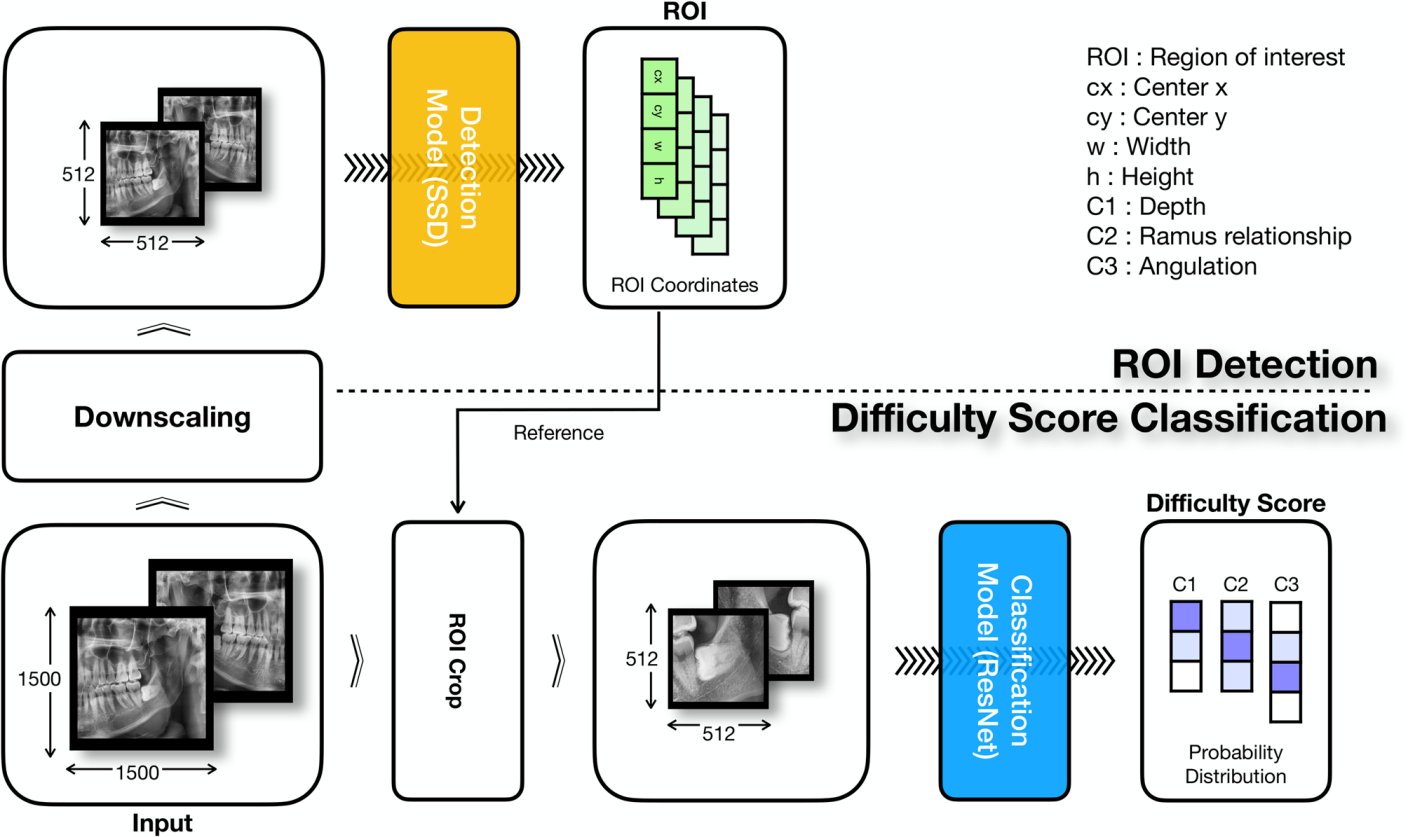
Entire diagnosis process adopted in this study.

Our proposed diagnosis model, as illustrated in Fig. 5, can be divided into two phases: ROI detection and a difficulty index classification. First, we find an ROI that includes the region of the mandibular third molar using the object detection model. The detection model outputs the coordinates of the ROI, and we crop the ROI from the original image. Subsequently, the classification model classifies the cropped ROI into the appropriate index according to the PDS.

1. *ROI detection* We used the SSD^18^ as the ROI detection model. The size of the input image was downscaled from 1500 × 1500 to 512 × 512 because the original was too large to be used as an input. We used a VGG16 pretrained on the ImageNet dataset as a backbone network of the detection model. In addition, the mandibular third molar region, which is the ROI, has less variability in terms of scale and proportion than that of ordinary objects. Therefore, by removing the less useful default boxes, the overall computation and processing time could also be reduced.

The aforementioned model aims to find a suitable region for a score evaluation. Thus, to train this model, the target data including the region information need to be identified. In this study, we determined the suitable scope while simultaneously estimating the difficulty score.

2. *Difficulty classification* An image cropped from the ROI of the original image, as predicted by the detection model, is used as the input. We proposed applying a classification model because the scores did not match the gradual variation in the radiographic image. The backbone network of the classification model used in our study was an ResNet-34^21^ pretrained on the ImageNet dataset. Feature maps extracted by the backbone network were converted into feature vectors through global average pooling. Next, three fully connected layers classified the feature vectors into the appropriate PDS values.

### Training details

We used the stochastic gradient descent as an optimizer with a learning rate of 0.01, weight decay of 0.9, mini-batch size of 32, and momentum of 0.9. We divided the learning rate by 10 for 250 iterations. In addition, we used gradient clipping to ensure that the training remained stable. The detection model loss is the weighted sum between the localization loss and the confidence loss. The localization loss is Smooth L1 loss, and the confidence loss is SoftMax cross-entropy loss.

### Statistical analysis

A statistical analysis was conducted by calculating the accuracy, sensitivity, and specificity as listed in Table 1. In addition, the RMSE between the predicted and true Pederson scores was calculated to analyze whether our proposed model was able to predict the mandibular extraction difficulty similarly to that of the experts. Given the true positives (TP), true negatives (TN), false positives (FP), and false negatives (FN), the sensitivity and specificity were calculated using the following equations for each class.

$$Sensitivity = \frac{TP}{{TP + FP}}$$

$$Specificity = \frac{TN}{{TN + FN}}$$

The accuracy and Cohen’s kappa score (k) were calculated as follows:

$$Accuracy \left( {{\text{P}}_{o} } \right) = \frac{\sum TP}{{\sum TP + TN}},$$

$$k = \frac{{{\text{P}}_{o} - {\text{P}}_{e} }}{{1 - {\text{P}}_{e} }},$$

where ${\text{P}}_{o}$ is the observed agreement, which is the same as the accuracy, and ${\text{P}}_{e}$ is the expected agreement, which is due to chance. In addition, ${\text{P}}_{e}$ is given by $\sum \left( {TP + TN} \right)\left( {TP + FP} \right)/\left( {\sum TP + TN} \right)^{2} .$

We can also calculate the RMSE as follows:

$${\text{RMSE}} = \sqrt {\frac{1}{N}\mathop \sum \limits_{n}^{N} \left( {\hat{y}_{n} - y_{n} } \right)^{2} } ,$$

where ${\text{N}}$, $\hat{y}$, and $y$ are the number of samples, prediction value, and target value, respectively.

### Ethical approval and informed consent

This study was conducted in accordance with the guidelines of the World Medical Association Helsinki Declaration for biomedical research involving human subjects and was approved by the Institutional Review Board of Daejeon Dental Hospital, Wonkwang University (W2004/001-001). The IRB waived the need for individual informed consent, either written or verbal, from the participants owing to the non-interventional retrospective design of this study and because all data were analyzed anonymously.

## Data Availability

The data used in this study can be made available if needed within the regulation boundaries for data protection.

## References

[1] Krois J (2019). Deep learning for the radiographic detection of periodontal bone loss. Sci. Rep..

[2] Fernandez Rojas R, Huang X, Ou KL (2019). A Machine learning approach for the identification of a biomarker of human pain using fNIRS. Sci. Rep..

[3] Kwak GH (2020). Automatic mandibular canal detection using a deep convolutional neural network. Sci. Rep..

[4] Jaskari J (2020). Deep learning method for mandibular canal segmentation in dental cone beam computed tomography volumes. Sci. Rep..

[5] Kim DW (2019). Deep learning-based survival prediction of oral cancer patients. Sci. Rep..

[6] Hallac RR, Lee J, Pressler M, Seaward JR, Kane AA (2019). Identifying ear abnormality from 2D photographs using convolutional neural networks. Sci. Rep..

[7] Chang HJ (2020). Deep learning hybrid method to automatically diagnose periodontal bone loss and stage periodontitis. Sci. Rep..

[8] Lee JH, Kim DH, Jeong SN (2020). Diagnosis of cystic lesions using panoramic and cone beam computed tomographic images based on deep learning neural network. Oral Dis..

[9] Choi J, Eun H, Kim C (2018). Boosting proximal dental caries detection via combination of variational methods and convolutional neural network. J. Signal Process. Syst..

[10] Mallishery S, Chhatpar P, Banga KS, Shah T (2019). & Gupta, P.

[11] Yu, H. J. *et al.* Automated skeletal classification with lateral cephalometry based on artificial intelligence. *J. Dent. Res.* 22034520901715 (2020). 10.1177/0022034520901715 31977286

[12] Jeong SH (2020). Deep learning based discrimination of soft tissue profiles requiring orthognathic surgery by facial photographs. Sci. Rep..

[13] Lee KS, Jung SK, Ryu JJ, Shin SW, Choi J (2020). Evaluation of transfer learning with deep convolutional neural networks for screening osteoporosis in dental panoramic radiographs. J. Clin. Med..

[14] Hiraiwa T (2019). A deep-learning artificial intelligence system for assessment of root morphology of the mandibular first molar on panoramic radiography. Dentomaxillofac. Radiol..

[15] Renton T, Smeeton N, McGurk M (2001). Factors predictive of difficulty of mandibular third molar surgery. Br. Dent. J..

[16] Gbotolorun OM, Arotiba GT, Ladeinde AL (2007). Assessment of factors associated with surgical difficulty in impacted mandibular third molar extraction. J. Oral Maxillofac. Surg..

[17] Yuasa H, Kawai T, Sugiura M (2002). Classification of surgical difficulty in extracting impacted third molars. Br. J. Oral Maxillofac. Surg..

[18] Liu, W. *et al.* SSD: Single Shot MultiBox detector, in *European Conference on Computer Vision* (2016).

[19] Suphangul S, Rattanabanlang A, Amornsettachai P, Wongsirichat N (2016). Dimension distortion of digital panoramic radiograph on posterior mandibular regions. M. Dent. J..

[20] Vinayahalingam S, Xi T, Bergé S, Maal T, de Jong G (2019). Automated detection of third molars and mandibular nerve by deep learning. Sci. Rep..

[21] He, K., Zhang, X., Ren, S. & Sun, J. Deep residual learning for image recognition in *IEEE Conference on Computer Vision and Pattern Recognition (CVPR)* 770–778 (2016).

